# Transcription factor Krüppel-like factor 5-regulated N-myc downstream-regulated gene 2 reduces IL-1β-induced chondrocyte inflammatory injury and extracellular matrix degradation

**DOI:** 10.1080/21655979.2021.1971483

**Published:** 2021-09-23

**Authors:** Xiaoliang Mei, Hao Din, Jianning Zhao, Jian Tong, Wei Zhu

**Affiliations:** a Department of Orthopedics, Taizhou Clinical Medical School of Nanjing Medical University (Taizhou People’s Hospital), Taizhou, People’s Republic of China; b Department of Orthopedics, Jinling Hospital, Jinling Clinical Medical College of Nanjing Medical University, Nanjing, People’s Republic of China

**Keywords:** Osteoarthritis, NDRG2, chondrocyte apoptosis, inflammatory response, extracellular matrix degradation

## Abstract

Previous research has identified N-myc downstream-regulated gene 2 (NDRG2) as one of the differentially expressed genes common to rat models of osteoarthritis (OA) and human OA. The purpose of this study was to investigate the role of NDRG2 in OA. In this study, an in vitro OA model was constructed by challenging ATDC5 chondrocytes with 10 ng/ml IL-1β. After transfection of pcDNA3.1(+)/NDRG2, qPCR and western blot were performed to assay NDRG2 expression. The analyses of cell viability, apoptosis and inflammatory molecule expression were employed respectively by CCK-8, TUNEL and ELISA. The protein expression related to apoptosis, inflammation or extracellular matrix (ECM) degradation was detected by western blot. The binding of Krüppel-like factor 5 (KLF5) to NDRG2 promoter was verified by means of dual-luciferase reporter assay. After overexpression of both NDRG2 and KLF5 in IL-1β-stimulated ATDC5 chondrocytes, corresponding assays were performed to examine cell viability, apoptosis, inflammatory response and ECM degradation. In ATDC5 chondrocytes challenged with IL-1β, NDRG2 expression was much lower than that in the control group, whereas it’s overexpression helped restored cell viability and reduce cell apoptosis, inflammatory response and ECM degradation. It was also observed that KLF5 expression was decreased in IL-1β-stimulated ATDC5 chondrocytes, and that KLF5 bound to the NDRG2 promoter. Importantly, overexpressing KLF5 could reverse the protective effect of NDRG2 overexpression on IL-1β-stimulated ATDC5. Overall, NDRG2 could be transcriptionally regulated by transcription factor KLF5 and may play a protective role against chondrocyte the inflammatory response and ECM degradation in OA.

## Introduction

Osteoarthritis (OA) refers to the gradual degeneration of the bone and joint starting from injury of the articular cartilage that develops into lesions of the entire articular tissue, such as irreversible cartilage degradation, fibrosis, fracture and defect of the articular surface [1]. According to the statistics of China Health and Retirement Longitudinal Study (CHARLS), the prevalence of symptomatic knee OA in people over 45 years old is approximately 8.1%, with the figure higher in women than men, and the prevalence of symptomatic hip OA is 1.1% in men and 0.9% in women [2]. Worldwide, OA remains a global health issue affecting over 240 million people, predominantly the elderly [3]. The general incidence and prevalence of OA are continuously on the rise each year, causing long-term economic and psychological burden on the patients [4]. Evidence is emerging to suggest that inflammation-related pain has gradually been a major clinical challenge in terms of OA treatment [5]. However, due to lack of effective treatment methods, an urgent requirement for the identification of therapeutic targets is of great necessity for OA therapies [6].

A study comparing the molecular pathophysiological pathways in rat models of OA and human osteoarthritic cartilages has identified 20 common genes that were differentially expressed, including N-myc downstream-regulated gene 2 (NDRG2) [7]. Later on, through microarray expression profiling, 16 of the same set of genes showed dysregulation at any point in time in the cartilage tissues of mice with the medial meniscus surgery-induced OA [8]. NDRG2 again ranks among the 16 differentially expressed genes, implying its potential role in OA. NDRG2 has been recently reported to participate in osteoblast differentiation and calcification in osteoporosis in part through the JAK3/STAT3 signaling pathway [9]. It also possesses an anti-inflammatory function in glioma cell lines by activating IL-6/JAK2/STAT3 [10]. The present study focuses on analyzing whether and how NDRG2 affects chondrocyte apoptosis, inflammatory response and extracellular matrix degradation in OA and revealing the potential mechanism.

We speculated that NDRG2 and KLF5 play important roles in OA. In this study, we aimed to illustrate the roles and the mechanism of NDRG2 and KLF5 in IL-1β-induced ATDC5. And we want to provide a theoretical basis for targeted treatment of OA.

## Materials and methods

### Cell culture and treatment

ATDC5 chondrocytes obtained from Beina Biological Technology Co., LTD were added into RPMI 1640 medium (Gibco) with 10% fetal bovine serum (Gibco) and cultured in an incubator with 5% CO_2_ at 37°C. IL-1β (Acro Biosystems, Beijing, China) at the concentrations of 1, 3, 10, or 30 ng/ml was chosen to treat ATDC5 cells, following the method previously reported by Chien et al. [11].

### Cell transfection

pcDNA3.1(+)/NDRG2 (Ov-NDRG2), pcDNA3.1(+)/KLF5 (Ov-KLF5), empty pcDNA3.1(+) vectors (Ov-NC), wild-type NDRG2 (NDRG2-WT) and mutant NDRG2 (NDRG2-MUT) were constructed by GenePharma (Shanghai, China). Transfection of these plasmids in different groups was performed in accordance with the instructions of Lipofectamine 3000 (Invitrogen) at 37°C for 48 h according to the manufacturer’s protocol. RT-qPCR and western blot were used to detect the transfection efficiency 48 h after transfection. ATDC5 cells were transfected with Ov-NDRG2 and Ov-KLF5 and then treated with IL-1β. The cells were divided into control, IL-1β, IL-1β + Ov-NDRG2, IL-1β + Ov-NDRG2 + Ov-NC and IL-1β + Ov-NDRG2 + Ov-KLF5.

### Real-time quantitative PCR (qPCR)

Total RNA was extracted by means of TRIzol® reagent (Thermo Fisher Scientific, Inc.) according to the suppliers’ instructions and was reverse-transcribed into cDNA using Hifair® III 1st Strand cDNA Synthesis SuperMix for qPCR (Yeasen, Shanghai, China). cDNA (1 μL) was taken as the template for real-time PCR, and the reaction conditions were: 95°C for 10 min, 95°C for 15 s, 60°C for 30 s, 72°C for 30 s, and total 35 cycles. The relative quantity of gene expression was calculated with 2^–∆∆Ct^ method [12]. The sequences of the primers were as follows: NDRG2 forward, 5ʹ- CACTCCAGTGACAGCACCTCT-3ʹ and reverse 5ʹ- GGCTCCAACACCAACTCC AATT-3ʹ; KLF5 forward, 5ʹ-GGACTCATACGGGCGAGAAG-3ʹ and reverse, 5ʹ- TAAAGGATGGCAGAGCGGAC-3ʹ; and GAPDH forward, 5ʹ-ATTGTCAGCAATGCATCCTG-3ʹ and reverse 5ʹ-GTAGGCCATGAGGTCCACCA-3ʹ.

### Western blot (WB)

After the cells in different groups were digested, centrifuged and lysed, the total proteins were isolated for detection of protein concentration. 30 μL of protein samples was taken for electrophoresis in 12% SDS-PAGE gel. After electrophoresis, the protein was transferred to PVDF membrane by wet transfer method. Primary antibodies (1:1500) were added to the membrane blocked by 50 g/L of skim milk and incubated overnight at 4°C. The corresponding horseradish peroxidase labeled secondary antibody (1:1 000) was added on the next day for 1 h before ECL color development. The relative quantity of protein expression in different groups was calculated using Image J software. Primary antibodies information were as follows: anti-NDRG2 (ab174850, Abcam), anti-Bcl-2 (ab32124, Abcam), anti- Bax (ab182733, Abcam), anti- cleaved caspase 3 (ab214430, Abcam), anti- caspase 3 (ab184787, Abcam), anti- cleaved PARP (ab225751, Abcam), anti- PARP (ab191217, Abcam), anti- TNF-α (ab183218, Abcam), anti- IL-6 (ab259341, Abcam), anti- p-NF-κB p65 (ab239882, Abcam), anti- NF-κB p65 (ab207297, Abcam), anti- Cox-2 (ab179800, Abcam), anti- MMP3 (ab52915, Abcam), anti- MMP13 (PA5-116,704, Thermo Fisher Scientific), anti- ADAMTS-4 (ab185722, Abcam), anti- Collagen II (ab7778, Abcam), anti- KLF5 (ab137676, Abcam), anti-GAPDH (ab8245, Abcam).

### Analysis of cell viability by CCK-8

ATDC5 cells (2 x 10^4^ cells/mL) were cultured in 96-well plates at 37°C. After transfection, the cells were treated with IL-1β in each group and the 10 μL of CCK-8 solution (HanBio, Shanghai, China) was added for incubation for 1 h. The optical density in each group was measured with a microplate reader at 450 nm wavelength [13].

### TUNEL assay of cell apoptosis

The cell nuclei were stained with DAPI following the instructions of the One-Step TUNEL Assay Kit (Beyotime, Nanjing, China) and then photographed with a fluorescent microscope. The number of DAPI-positive cells and total cell number were counted, and their ratio equals the apoptosis rate [14].

### Detection of pro-inflammatory cytokines by ELISA

The levels of TNF-α and IL-6 were detected by Mouse Interleukin-6/Tumor Necrosis Factor-α Enzyme-Linked ImmunoSorbent Assay Kits (Beyotime, Nanjing, China) in accordance with the manufacturer’s protocols. The absorbance (A450) was detected by a microplate reader.

### Dual-luciferase reporter assay

The luciferase reporter assay was performed to examine NDRG2-KLF5 binding affinity. ATDC5 cells were co-transfected with NDRG2-WT/MUT and Ov-KLF5/NC, as described above. 48 h later, the supernatant was taken for measurement of the firefly and Renilla luciferase activities. The relative luciferase activities equals the ratio of the firefly to Renilla luciferase activities.

### Statistical analysis

All assays were carried out in triplicate, and the results were analyzed by SPSS 20.0. The numerical data are expressed as the mean ± SD. All data are normally distributed. Student t test was used to compare the mean values of two independent samples, and one-way analysis followed by the post-hoc analysis of variance was used to compare the mean values of multiple groups. The difference with a P value of less than 0.05 is considered statistically significant. Each experiment repeated at least three times.

## Results

### [1] NDRG2 expression decreased in IL-1β-stimulated ATDC5

**Figure 1.**
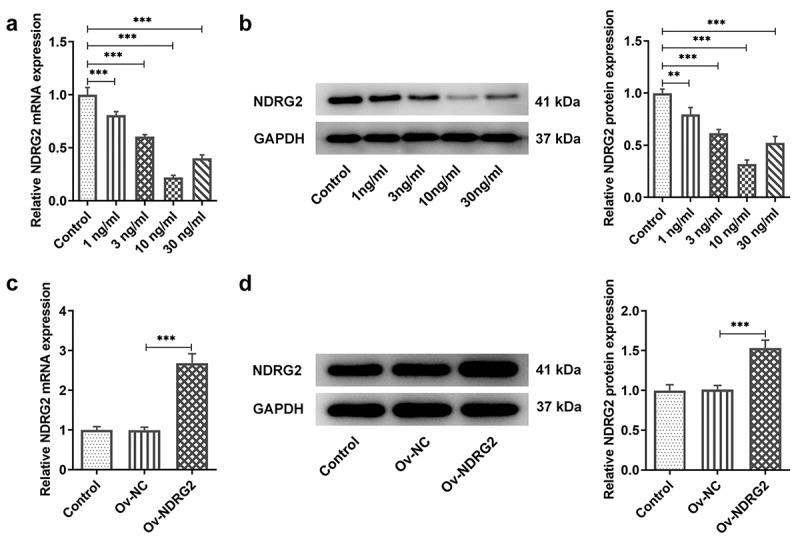
NDRG2 expression decreased in IL-1β-stimulated ATDC5. NDRG2 expression decreased in IL-1β-stimulated ATDC5 (a and b) NDRG2 expression in ATDC5 cells treated with different concentrations of IL-1β, detected by qPCR and WB. **P < 0.01, ***P < 0.001 (c and d) NDRG2 expression in control-treated, OV-NC- or Ov-NDRG2-transfected ATDC5, Ov-NC or Ov-NDRG2, detected by qPCR and WB. ***P < 0.001

We first detected the expression of NDRG2. Compared to the control group, ATDC5 chondrocytes treated with IL-1β at the concentration of 1, 3, 10 and 30 ng/ml expressed lower levels of NDRG2, with 10 ng/ml leading to the lowest NDRG2 level (Figure 1. A and B). It suggested that NDRG2 expression was decreased in IL-1β-stimulated ATDC5. 10 ng/ml of IL-1β was therefore selected to establish the in vitro OA model. To perform the gain-of-function experiment on NDRG2, Ov-NDRG2 was transfected into ATDC5. The overexpression efficacy was confirmed by the results of qPCR and WB (Figure 1. C and D).

### [2] NDRG2 overexpression improved the viability and inhibited the apoptosis of IL-1β-stimulated ATDC5

**Figure 2.**
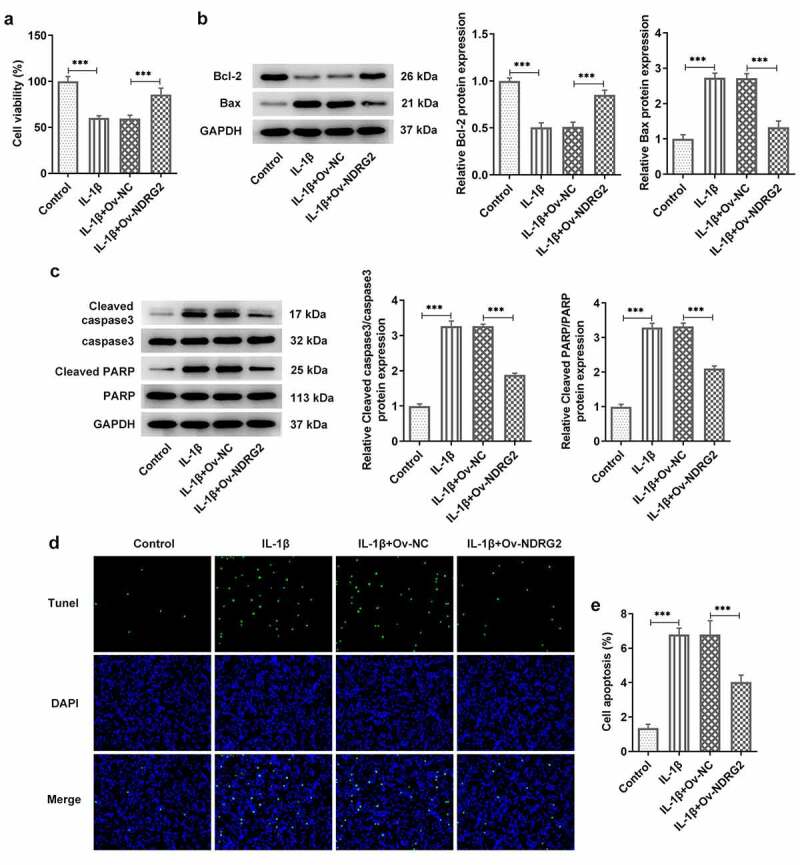
NDRG2 overexpression improved the viability and inhibited the apoptosis of IL-1β-stimulated ATDC5. NDRG2 overexpression improved the viability and inhibited the apoptosis of IL-1β-stimulated ATDC5 (a) The viability of IL-1β-stimulated ATDC5 cells analyzed with CCK-8 after transfection of Ov-NC or Ov-NDRG2. ***P < 0.001 (b and c) The expression of anti-apoptotic Bcl-2 and apoptosis-related proteins in IL-1β-stimulated ATDC5 cells after transfection of Ov-NC or Ov-NDRG2, detected by WB. ***P < 0.001 (d and e) TUNEL-positive cells under IL-1β stimulation after transfection of Ov-NC or Ov-NDRG2, and the corresponding apoptosis rate. ***P < 0.001

We examined the effects of NDRG2 on cell viability and apoptosis and found that the viability of ATDC5 cells was reduced by IL-1β stimulation but noticeably improved after the transfection of Ov-NDRG2 into IL-1β-stimulated ATDC5 (Figure 2. A). In addition, an upregulation of the levels of apoptosis markers Bax, cleaved caspase 3 and cleaved PARP/PARP and a downregulation of Bcl-2 were observed in IL-1β-stimulated ATDC5, while NDRG2 overexpression reversed these trends to a large extent (Figure 2. B and C). The results of TUNEL assay demonstrated increased number of TUNEL-positive cells in the IL-1β group and decreased number of those in the IL-1β+Ov-NDRG2 group, indicating that IL-1β-induced cell apoptosis was alleviated by NDRG2 overexpression (Figure 2. D-E). Therefore, it was ascertained that NDRG2 overexpression improved the viability and inhibited the apoptosis of IL-1β-stimulated ATDC5.

### [3] NDRG2 overexpression inhibited IL-1β-induced inflammatory cytokine release and the expression of ECM degradation proteins

**Figure 3.**
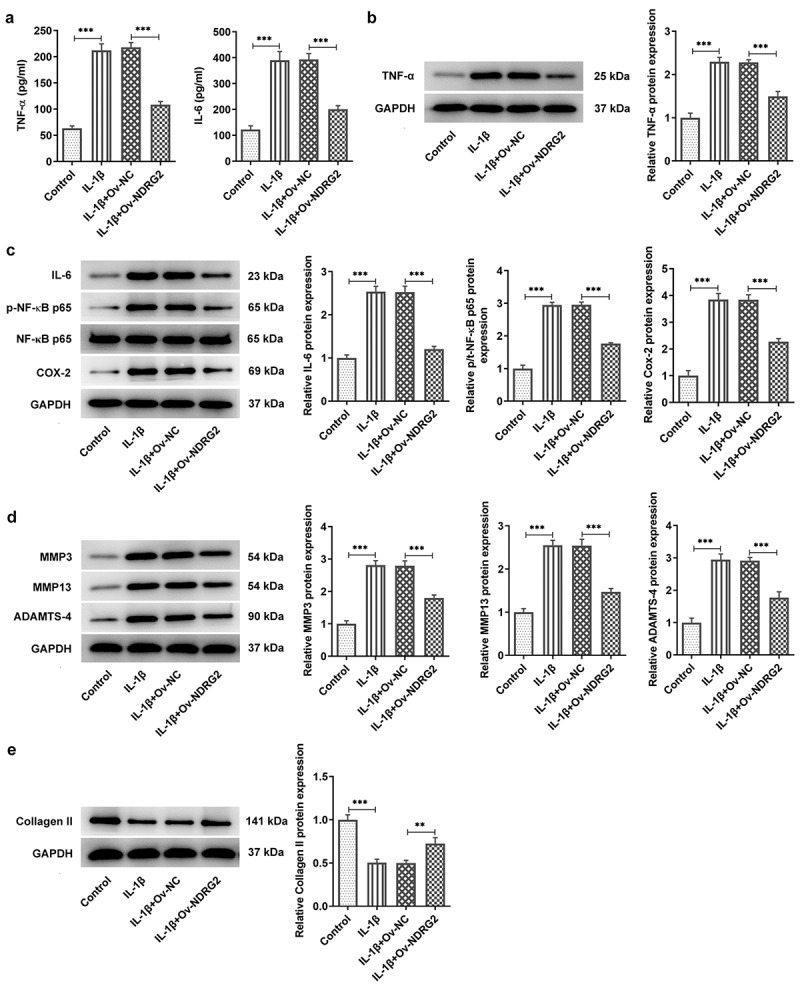
NDRG2 overexpression inhibited IL-1β-induced inflammatory cytokine release and ECM degradation. NDRG2 overexpression inhibited IL-1β-induced inflammatory cytokine release and ECM degradation (a-c) The levels of pro-inflammatory cytokines TNF-α, IL-6, p-NF-κB p65 and Cox-2 in IL-1β-stimulated ATDC5 cells after transfection of Ov-NC or Ov-NDRG2, detected by ELISA or WB. ***P < 0.001 (d and e) ECM-degrading enzymes and collagen II expression assayed by WB in IL-1β-stimulated ATDC5 cells after transfection of Ov-NC or Ov-NDRG2. **P < 0.01, ***P < 0.001

It was found through ELISA and WB that treatment with IL-1β provoked the release of pro-inflammatory TNF-α, IL-6, p-NF-κB p65 and Cox-2 in ATDC5 cells, and that NDRG2 overexpression suppressed IL-1β-induced high levels of inflammatory cytokines (Figure 3. A, B and C). MMP3, MMP13 and ADAMTS-4 are the ECM protein component-degrading enzymes [15,16], which were shown to be upregulated in IL-1β-stimulated ATDC5 and were downregulated by NDRG2 overexpression (Figure 3. D and E). Collagen II, on the other hand, was downregulated in IL-1β-stimulated ATDC5 and was upregulated by NDRG2 overexpression. These results revealed the inhibitory effect of NDRG2 overexpression on IL-1β-induced inflammatory cytokine release and ECM degradation-related proteins.

### [4] Transcriptional inhibition of NDRG2 by KLF5

**Figure 4.**
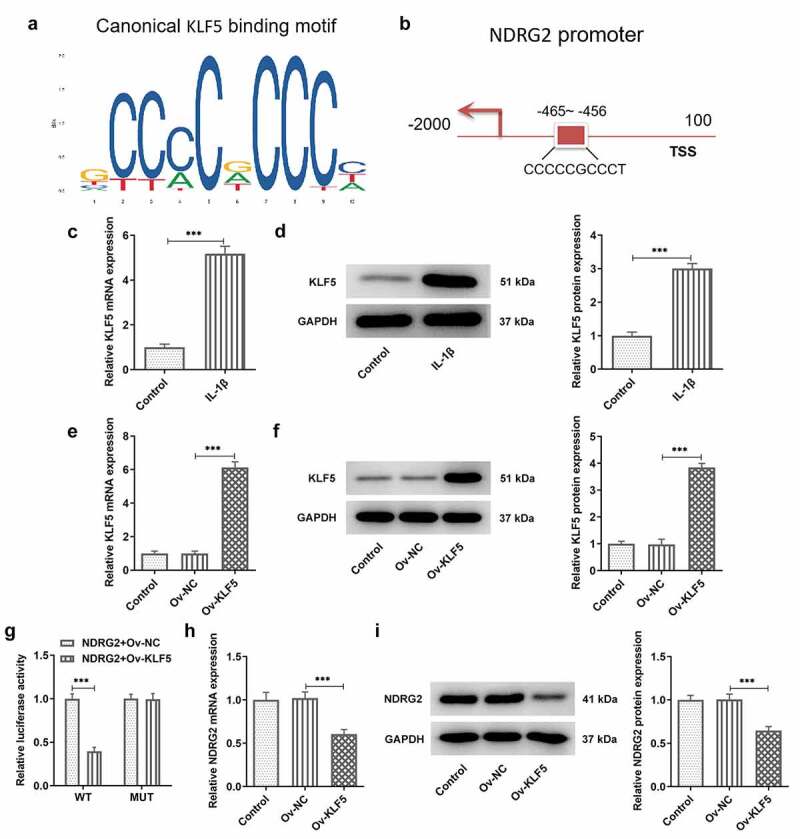
Transcriptional inhibition of NDRG2 by KLF5. Transcriptional inhibition of NDRG2 by KLF5 (a and b) Canonical KLF5 binding motif and NDRG2 promoter sequences, predicted by JASPAR. (c and d) KLF5 expression in control or IL-1beta-treated ATDC5 cells, detected by qPCR and WB. ***P < 0.001 (e and f) KLF5 expression after transfection of Ov-KLF5, detected by qPCR and WB. ***P < 0.001 (g) Relative luciferase activity in cells co-transfected with NDRG2-WT/MUT and Ov-KLF5/NC. (h and i) NDRG2 expression after transfection of Ov-KLF5, detected by qPCR and WB. ***P < 0.001

JASPAR database predicted the binding sites of KLF5 on NDRG2 promoter (Figure 4. A and B). In ATDC5 chondrocytes challenged with IL-1β, qPCR and WB detected a marked increase in the mRNA and protein expression of KLF5 (Figure 4. C and D). Subsequently, qPCR and WB confirmed the overexpression efficacy of the transfection of Ov-KLF5 into ATDC5 (Figure 4. E and F). Furthermore, the luciferase activity in cells co-transfected with NDRG2-WT and Ov-KLF5 showed a clear reduction compared to the Ov-NC group, while the luciferase activity showed no difference between the NDRG2-MUT+Ov-NC group and the NDRG2-MUT+Ov- KLF5 group (Figure 4. G). In ATDC5 cells transfected with Ov-KLF5, the expression of NDRG2 was reduced by contrast with the Ov-NC group (Figure 4. H and I). These results confirmed the binding interaction between NDRG2 and KLF5 and the transcriptional inhibition of NDRG2 by KLF5.

### [5] KLF5 overexpression reversed the improvement of NDRG2 overexpression on the viability and the apoptosis of IL-1β induced ATDC5

**Figure 5.**
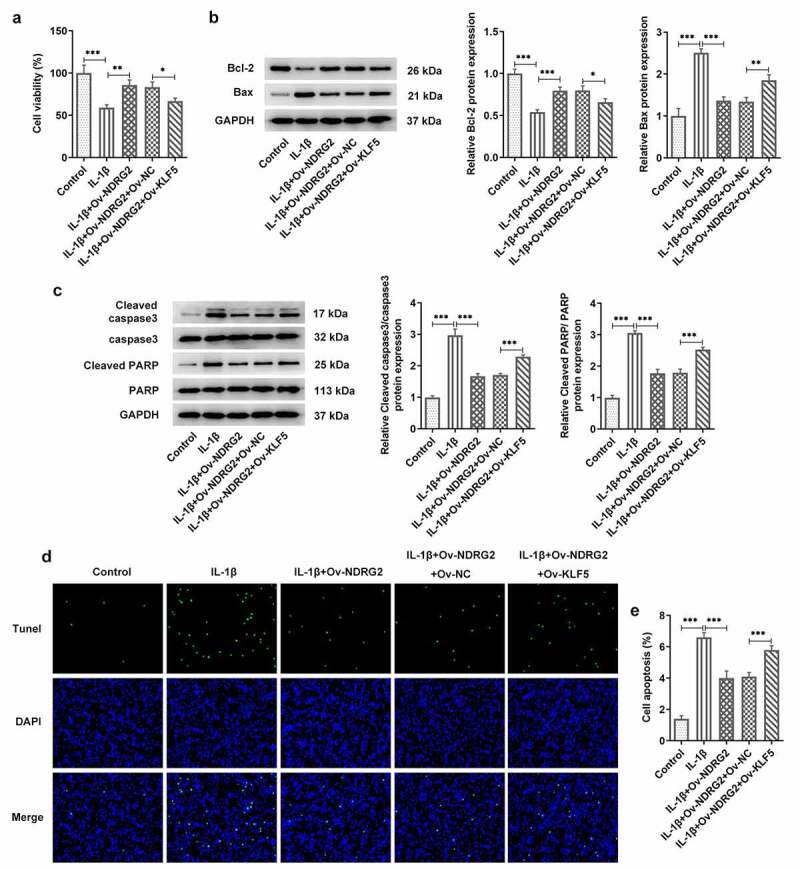
KLF5 overexpression reversed the improvement of NDRG2 overexpression on the viability and the apoptosis of IL-1β induced ATDC5. KLF5 overexpression reversed NDRG2 overexpression-attenuated ATDC5 viability loss and apoptosis induced by IL-1β (a) The viability of IL-1β-stimulated ATDC5 cells analyzed with CCK-8 after transfection of Ov-NC, Ov-NDRG2, Ov-NDRG2+ Ov-NC or Ov-NDRG2+ Ov-KLF5. *P < 0.05, **P < 0.01, ***P < 0.001 (b and c) The expression of anti-apoptotic Bcl-2 and apoptosis-related proteins in IL-1β-stimulated ATDC5 cells after transfection of Ov-NC, Ov-NDRG2, Ov-NDRG2+ Ov-NC or Ov-NDRG2+ Ov-KLF5, detected by WB. *P < 0.05, **P < 0.01, ***P < 0.001 (d and e) TUNEL-positive cells under IL-1β stimulation after transfection of Ov-NC, Ov-NDRG2, Ov-NDRG2+ Ov-NC or Ov-NDRG2+ Ov-KLF5, and the corresponding apoptosis rate. ***P < 0.001

Next, we further discussed the mechanism of KLF5 and NDRG2 in OA. Compared to IL-1β-stimulated ATDC5 transfected with Ov-NDRG2, ATDC5 cell viability was decreased after co-transfection of Ov-NDRG2 and Ov-KLF5 (Figure 5. A). KLF5 overexpression also decreased the expression of Bcl-2 and increased that of Bax, cleaved caspase 3 and cleaved PARP/PARP, which reversed the effects of NDRG2 overexpression on the levels of these proteins in IL-1β-stimulated ATDC5 chondrocytes (Figure 5. B and C). Additionally, an increase in the number of TUNEL-positive cells was observed in IL-1β-stimulated ATDC5 co-transfected with Ov-NDRG2 and Ov-KLF5, in comparison with the IL-1β+Ov-NDRG2+ Ov-KLF5 group (Figure 5. D and E). Therefore, NDRG2 overexpression-attenuated ATDC5 viability loss and apoptosis induced by IL-1β could be reversed by KLF5 overexpression.

### [6] KLF5 overexpression reversed NDRG2 overexpression-alleviated inflammatory cytokine release and the expressions of ECM degradation proteins induced by IL-1β

**Figure 6.**
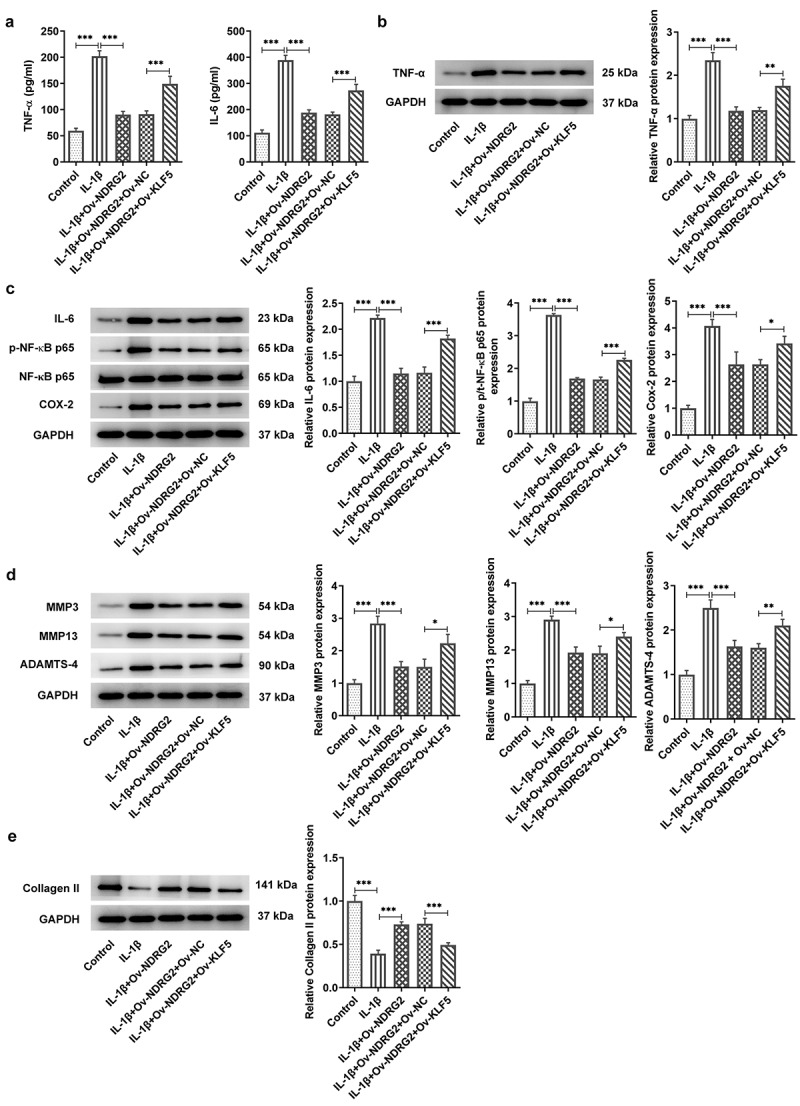
KLF5 overexpression reversed NDRG2 overexpression-alleviated inflammatory cytokine release and ECM degradation induced by IL-1β. KLF5 overexpression reversed NDRG2 overexpression-alleviated inflammatory cytokine release and ECM degradation induced by IL-1β (a-c) The levels of pro-inflammatory cytokines TNF-α, IL-6, p-NF-κB p65 and Cox-2 in IL-1β-stimulated ATDC5 cells after transfection of Ov-NC, Ov-NDRG2, Ov-NDRG2+ Ov-NC or Ov-NDRG2+ Ov-KLF5, detected by ELISA or WB. *P < 0.05, **P < 0.01, ***P < 0.001 (d and e) ECM-degrading enzymes and collagen II expression assayed by WB in IL-1β-stimulated ATDC5 cells after transfection of Ov-NC, Ov-NDRG2, Ov-NDRG2+ Ov-NC or Ov-NDRG2+ Ov-KLF5. *P < 0.05, **P < 0.01, ***P < 0.001

In addition to changes in ATDC5 cell viability and apoptosis, KLF5 overexpression led to upregulated levels of pro-inflammatory cytokines TNF-α, IL-6, p-NF-κB p65 and Cox-2 (Figure 6. A and B). KLF5 overexpression also increased the expression of ECM degradation-related MMP3, MMP13 and ADAMTS-4 levels and decreased that of collagen II in IL-1β-stimulated ATDC5 cells, which offset the effects of NDRG2 overexpression on IL-1β-induced ECM degradation (Figure 6. D and E). These results indicated that KLF5 overexpression reversed NDRG2 overexpression-alleviated inflammatory cytokine release and ECM degradation-related proteins induced by IL-1β.

## Discussion

OA is a common chronic joint disease characterized by cartilage destruction, subchondral bone reconstruction and synovial inflammation [17,18]. The occurrence of these characteristics are primarily attributed to the imbalance between the anabolism and catabolism in the articular cartilage, especially the increase in catabolism [19]. Pro-inflammatory molecules, such as IL-1β, TNF-α and IL-6, are key mediators of this metabolic dyshomeostasis as they can increase the gene expression of matrix-degrading enzymes, thereby facilitating cartilage ECM degradation and promoting OA progression [20].

The fragments generated upon ECM degradation stimulate the release of pro-inflammatory cytokines, leading to exacerbated synovial inflammation and ultimately cartilage destruction [21]. On the other hand, these cytokines also blunt the synthesis of cartilage ECM proteins [22]. In addition, in several studies, IL-1β was used to stimulate chondroprogenitor ATDC5 cells to simulate inflammation in vitro, thus establishing OA in vitro model [23–25]. In the present study, the challenge of IL-1β in ATDC5 chondrocytes resulted in decreased cell viability and increased cell apoptosis, elevated levels of pro-inflammatory cytokine secretion and ECM-degrading enzyme expression, suggesting successful establishment of the in vitro OA model.

NDRG2, along with 19 other genes, has been reported to act as a differentially expressed gene common to rat models of OA and human osteoarthritic cartilages [7]. Microarray expression profiling of the same set of genes has illustrated 16 dysregulated genes in the cartilage tissues of mice with DMM surgery-induced OA, of which NDRG2 is a member [8]. In a destabilized medial meniscus surgery-induced mouse OA model, NDRG2 was identified as a key biomarker and modulator of OA [26]. The latest evidence shows that rheumatoid arthritis tissues express comparatively low levels of NDRG2 and that silencing NDRG2 promotes the proliferation and inflammation of fibroblast-like synoviocytes [27]. The present study also observed a significant decrease in the expression level of NDRG2 in the OA model group. In patients with ulcerative colitis, reduced NDRG2 expression is positively correlated with severe inflammation [28]. NDRG2 depletion led to destruction of adhesive junctive structures, reduced epithelial barrier function and increased intestinal epithelial permeability. However, NDRG2 has also been found to be upregulated after proinflammatory cytokine intervention in intestinal glia exposed to hypoxia-glucose deprivation/reoxygenation [29]. In our study, upregulating NDRG2 levels in IL-1β-challenged ATDC5 cells improved cell viability while inhibited cell apoptosis and inflammatory responses. Furthermore, the absence of NDRG2 renders MMP-3 and MMP-9 active in astrocytes and increases the permeability of blood-brain barrier, exacerbating blood cell infiltration and brain damage following permanent focal cerebral ischemia [30]. Similar to this finding, the present study observed an alleviative effect of NDRG2 overexpression on ECM degradation by downregulating the protein expression of ECM-degrading enzymes MMP3, MMP13 and ADAMTS-4 and upregulating that of collagen II.

As regards the mechanisms of such functions of NDRG2, JASPAR database was consulted and predicted KLF5 as a potential transcription factor binding to the promoter of NDRG2. KLF5 expression has been considered to be meaningful in the occurrence and development of OA. A previous study on OA has shown that IL-1β-induced overexpression of EGR1 leads to activated expression of KLF5 and inhibited KLF5 ubiquitination, accelerating cartilage degeneration and inducing chondrocytes hypertrophy in vitro [31]. Here in this study, KLF5 was found to be highly expressed in IL-1β-stimulated chondrocytes and proved to be able to bind to the promoter region of NDRG2. In a cellular model of chronic obstructive pulmonary disease, KLF5 as a target of miR-145-5p, when suppressed by miR-145-5p upregulation, could potentially protect against cigarette smoke exposure-induced bronchial epithelial cell inflammation and inflammation-related apoptosis [32]. KLF5 has also been shown to be associated with the inflammatory response in intervertebral disc degeneration, as it is involved in IL-1β-activated NF-κB cascade by enhancing p65 phosphorylation and p65 acetylation [33]. Our results demonstrated that KLF5 overexpression could abate the protective effect of NDRG2 overexpression on ATDC5 viability and the alleviative effect on IL-1β-induced cell apoptosis and inflammation by promoting pro-inflammatory cytokine release. In an earlier study, increased expression of KLF5 and MMP-9 was found to be co-localized in the same cells of severely deformed cartilage in tibiofemoral joint of OA patients treated with surgery alone [34]. The severity of cartilage degeneration and vascular invasion in OA are probably related to the upregulated expression of the two proteins. Consistent with this finding, our results also found that KLF overexpression elevated the protein levels of ECM-degrading MMP3, MMP13 and ADAMTS-4, which reversed the ameliorative effect of NDRG2 overexpression on IL-1β-induced ECM degradation.

There are some limitations in our experiment. We only selected one cell line for the experiment, and in the next experiment, we will verify the experimental results of this paper in other cell lines. In addition, we will conduct in vivo studies to further verify the results.

## Conclusion

To sum up, the present study demonstrated that NDRG2 could be regulated by transcription factor KLF5 in ATDC5 chondrocytes and reduced IL-1β-induced inflammatory responses and ECM degradation proteins. Data in this study explain the potential role of NDRG2 in OA and indicate that NDRG2 may not only be a biomarker but also a promising therapeutic target in OA.

## Data Availability

The analyzed data sets generated during the present study are available from the corresponding author on reasonable request.

## Research highlights

KLF5 transcription regulates NDRG2 expression

KLF5 regulates NDRG2 to reduce inflammation in OA

KLF5 regulates NDRG2 to reduce ECM degradation in OA
